# Editorial: Development, metabolism, senescence and mechanotransduction of bone

**DOI:** 10.3389/fcell.2022.1103581

**Published:** 2022-12-08

**Authors:** Suryaji Patil, Lifang Hu, Changqi Zhu, Cory J. Xian, Airong Qian

**Affiliations:** ^1^ Lab for Bone Metabolism, Xi’an Key Laboratory of Special Medicine and Health Engineering, Key Lab for Space Biosciences and Biotechnology, Research Center for Special Medicine and Health Systems Engineering, NPU-UAB Joint Laboratory for Bone Metabolism, School of Life Sciences, Northwestern Polytechnical University, Xi’an, Shaanxi, China; ^2^ Ferris State University, Big Rapids, MI, United States; ^3^ Clinical and Health Sciences, University of South Australia, Adelaide, SA, Australia

**Keywords:** bone development, bone meta, bone mechanotransduction, bone senescence, bone disease

Bone is an integral part of the musculoskeletal system, providing physical scaffolding as well as an attachment surface for tendons and ligaments to link muscles and bones. Importantly, it is the site of hematopoiesis, which is responsible for the rejuvenation of blood and immune cell populations essential for healthy physiology (Salhotra et al., 2020). The extraordinary ability of bone to repair and restore itself throughout life is tightly regulated by the coordinated processes of bone formation/mineralization and bone resorption, which are mediated by two of the most important bone cells, osteoblasts and osteoclasts, respectively. The anabolic and catabolic pathways of these cells, such as BMP-Smad, Wnt/β-catenin, Notch, and Hedgehog, determine and influence their ability to repair bone. Thus, any change in these pathways can disrupt bone homeostasis and lead to bone disorders such as osteoporosis (Suzuki et al., 2020). As a result, it is critical to investigate the expression of molecules in diseased conditions of bone in order to understand their role, which may open up new avenues for therapeutic development. Shin et al. Used knockout mice to demonstrate the importance of TLE4 in bone homeostasis. *Tle4* deficiency may impair not only hematopoiesis but also skeleton calcification *via* osteoblast function and differentiation by downregulating alkaline phosphatase (ALP), runt-related transcription factor 2 (Runx2), and osteocalcin expression.

The bone is an endocrine and mechanosensing organ in addition to its regular functions. Mechanical stimuli induce the bone to express and release “osteokines,” such as osteocalcin, sclerostin, Dickkopf-related protein 1 (Dkk1), and fibroblast growth factor, which have an effect on other tissues (Gerosa and Lombardi, 2021). Its ability to sense sensory cues and forces, particularly mechanical stimulation, influences its development and facilitates adaptation to changing environments (Liang et al., 2021). Osteocytes are the most abundant endocrine bone cells that regulate bone remodeling through calcium and phosphate metabolism as well as mechanical stimulation. When exposed to mechanical stress, their ability to recognize mechanical stimuli directly and indirectly allows them to promote bone adaptation and formation *via* the mechanotransduction process in individual cells, between neighboring cells, and their microenvironments *via* cell junctions (Qin et al., 2020). One of such gap junctions, connexin 43 (Cx43), has been shown to play an important role in bone formation in response to mechanical loading. Researchers discovered impaired anabolic responses in transgenic mouse models that expressed dominant-negative Cx43 in osteocytes, as well as increased endosteal osteoclast activity (Zhao et al., 2022).

Hua et al. discovered that Cx43 regulates the transition of osteoblast to osteocyte. Its deletion can postpone the transition while increasing osteoclastogenesis *via* the receptor activator of nuclear factor kappa-B ligand (RANKL)/osteoprotegerin (OPG). Aside from genetic changes, there is growing evidence that epigenetic changes such as DNA methylation, post-translational modifications, and non-coding RNA expression in cells can influence gene expression and bone metabolism (Yang et al., 2020). Huang et al. Have comprehensively reviewed one such modification, m^6^A methylation in bone marrow mesenchymal stem cells (BMSC), osteoblasts, and osteoclasts. Their work summarizes the effects of m^6^A modification on cell proliferation, differentiation, and apoptosis in these cells and osteoporosis and suggests that m6A modification could be a new target for osteoporosis treatment.

While many available therapeutics are promising in terms of bone regeneration, their long-term application is frequently limited due to adverse side effects. According to Gong et al., recombinant human globular adiponectin (ADPN) could be used to repair bone fractures. The study’s findings indicated that ADPN administration could promote bone formation by increasing osteogenic differentiation and proliferation of BMSCs *via* the AdipoR1 receptor. Importantly, it may reduce the number of osteoclasts *via* the OPG/RANKL pathway and promote bone fracture healing. Traditional Chinese medicines and their derivatives have played important roles in a variety of diseases due to their lower side effects (Huang et al.). Yan et al. discovered that by activating the BMP2/Smad/Runx/Osterix signaling pathway,β -ecdysterone, a steroidal phytohormone with the same chemical structure as estrogen, can improve bone regeneration in a bone injury mouse model. However, key component groups and the mechanisms of action of the constituents present in such medicines remain a mystery. Liu et al. Described a novel bioinformatics model that was used to identify the components and mechanisms of Gushukang Granules (GSK), Xianling Gubao Capsules (XLGB), and Er-xian Decoction (EXD). The model identified key components as quercetin, isoliquiritigenin, rutaecarpine, isofraxidin, and secoisolariciresinol, with a possible mechanism targeting osteoclast differentiation, calcium signaling pathways, MAPK signaling pathways, and the PI3K-Akt signaling pathway.

The articles in this Research Topic present and discuss broader aspects of bone physiology, ranging from elucidating the roles of various molecules and forces on bone cell development and differentiation, to investigating various molecules as potential therapeutics, to elucidating the effect of bone on other tissues, to identifying and proposing novel molecules as targets for bone disorders. These articles provide the basis for future experimental works and even for clinical applications.
